# Recognizing atypical depression in patients with chronic pain syndromes in general hospitals

**DOI:** 10.1192/j.eurpsy.2026.10341

**Published:** 2026-07-31

**Authors:** K. Krysta

**Affiliations:** Department of Rehabilitation Psychiatry, Medical University of Silesia, Katowice, Poland

## Abstract

**Abstract:**

Recognizing atypical depression in patients with chronic pain syndromes remains a major clinical challenge in general hospital settings. Chronic pain is frequently accompanied by depressive symptoms, yet these symptoms often present in non-classical or “atypical” forms that may be overlooked or misattributed to somatic illness. Unlike typical major depression, atypical depression is characterized by mood reactivity, hypersomnia, increased appetite or weight gain, leaden paralysis, and heightened interpersonal sensitivity. In patients with chronic pain, these features may be masked by fatigue, sleep disturbances, and functional limitations related to the underlying medical condition, complicating accurate diagnosis.

In general hospitals, the focus on somatic treatment and the fragmentation of care between specialties further increase the risk of underrecognition. Patients may primarily report pain intensity, bodily discomfort, or treatment resistance, while affective symptoms remain unexpressed or are minimized. Neurobiological mechanisms shared by chronic pain and atypical depression—including dysregulation of inflammatory pathways, altered stress response systems, and changes in central pain processing—suggest a bidirectional relationship that worsens both conditions if left untreated.

Failure to identify atypical depression in this population is associated with poorer pain control, prolonged hospital stays, reduced adherence to medical treatment, and impaired functional recovery. Early recognition requires a high index of clinical suspicion, careful assessment of mood reactivity and behavioral patterns, and the use of screening tools adapted to medically ill populations. Close collaboration between internists, pain specialists, psychiatrists, and psychologists is essential.

Integrating routine mental health screening into general hospital care and promoting a biopsychosocial approach can improve diagnostic accuracy and therapeutic outcomes. Timely identification and targeted treatment of atypical depression may not only alleviate psychological distress but also enhance pain management, rehabilitation, and overall quality of life in patients with chronic pain syndromes.

**Disclosure of Interest:**

None Declared

